# Mindfulness Meditation According to the Satipatthana Sutta: A Single-Case Study With Participants as Collaborators

**DOI:** 10.1007/s12671-023-02160-1

**Published:** 2023-06-10

**Authors:** Peter Sedlmeier, Alica Beckel, Samuel Conrad, Jan Husmann, Luisa Kullrich, Rico Lange, Anna-Lena Müller, Alexandra Neumann, Teresa Schaaf, Ayla Schaub, Alexandra Tränkner, Bianca Witzel

**Affiliations:** Department of Psychology, Chemnitz University of Technology, 09107 Chemnitz, Germany

**Keywords:** Mindfulness meditation, Participants as collaborators, Single-case experimental design, Satipatthana sutta

## Abstract

**Objectives:**

We explored the effects of a mindfulness program based on the *satipatthana sutta* (instead of a contemporary Western program), with participants as collaborators, using a single-case experimental design. The main question was whether such a training has positive effects and, if so, whether and how the effects vary across participants and measures.

**Method:**

Participants chose the design (multiple baseline) and the measures to be repeatedly collected. Then they took part in a 6-week mindfulness training based on the satipatthana sutta; finally, they performed a preliminary data analysis of their own results. Reported are a selection of the visual analyses, intraindividual effect sizes (Tau-*U*), and the results of single-case meta-analyses over participants, as well as a multivariate graphical analysis of interindividual differences.

**Results:**

Substantial training effects were found on average and for the majority of participants for concentration, mind wandering, decentering, positive affect, and well-being. Effects were small for negative affect, and no effects were found for emotion regulation. However, interindividual variation was high, both in respect to specific measures and concerning the overall effect of the training program. Participants' motivation was found to be very high throughout the study.

**Conclusion:**

Our findings indicate that a traditional mindfulness program yields effects that are roughly comparable to the effects of secular mindfulness training regimens. Regarding participants as collaborators appears to have a strong motivating effect. And finally, the study demonstrates that using single-case experimental designs (instead of group designs) allows for a more fine-grained analysis of meditation effects. The high interindividual variation points to the possibility that the amount of unexplained variance in group studies is severely underestimated. Results from studies like the current one could have benefits for both theoretical advancement and custom-tailored assignment of specific forms of meditation to specific people with specific aims.

**Supplementary Information:**

The online version contains supplementary material available at 10.1007/s12671-023-02160-1.

In a typical study on the effects of mindfulness meditation, the practice is taught in a way that is only partially in accordance with how mindfulness meditation was, and is, practiced in traditional Buddhist approaches (Anālayo, 2019a; Davidson & Dahl, 2018; Giles, 2019; Jinpa, 2019; Shonin et al., 2015; van Gordon & Shonin, 2020; Waldron, 2019). Moreover, participants usually do not have a say in the specifics of the study, and the effects are almost always examined in some kind of group comparison. Here, we argue that (a) traditional Buddhist ways of practicing mindfulness meditation might be valuable alternatives to conventional (secular) approaches; (b) it might be worthwhile to regard participants as collaborators (and not as mere subjects); and (c) using single-case designs as alternatives to group designs might have several advantages.

From the contemporary literature on the topic, one can get the impression that "mindfulness meditation" (or "mindfulness") is a synonym for "meditation." The term is used for techniques employing focused attention and open monitoring (Hölzel et al., 2011), for collections of quite diverse meditation techniques from one (Ortner et al., 2007) or multiple (Santorelli et al., 2017) traditional backgrounds, but also for concentrative meditation (Zeidan et al., 2010), for simply attending to the breath (Zeidan et al., 2011), and for mantra meditation (Tanner et al., 2009). To be sure, there is no total agreement even among Buddhist scholars about what exactly mindfulness (*sati*) means in different traditional contexts (e.g., Amaro, 2015; Anālayo, 2019b; Gethin, 2011; Husgafvel, 2016; Levman, 2018; Sharf, 2014; Shonin et al., 2015). Sati does not even play the main role in most of the relatively recent and well-known forms of Vipassana meditation, a 19th-century movement from Myanmar (Anālayo, 2012; Cousins, 1996; Kornfield, 1977; Shaw, 2021).

Yet, there seems to be a consensus that the *satipatthana sutta* should be considered an authoritative textual basis for what constitutes the traditional practice of mindfulness. In this sutta, the Buddha first highlighted the importance of the practice of the satipatthanas—the four foundations of mindfulness (Anālayo, 2003, p. 3):

Monks, this is the direct path for the purification of beings, for the surmounting of sorrow and lamentation, for the disappearance of *dukkha* and discontent, for acquiring the true method, for the realization of *Nibbāna*, namely, the four *satipaṭṭhānas*.

Then he went on to describe the practice of the four satipatthanas (Anālayo, 2003, pp. 3–4):

What are the four? Here, monks, in regard to the body a monk abides contemplating the body, diligent, clearly knowing, and mindful, free from desires and discontent in regard to the world. In regard to feelings he abides contemplating feelings, diligent, clearly knowing, and mindful, free from desires and discontent in regard to the world. In regard to the mind he abides contemplating the mind, diligent, clearly knowing, and mindful, free from desires and discontent in regard to the world. In regard to *dhammas* he abides contemplating *dhammas*, diligent, clearly knowing, and mindful, free from desires and discontent in regard to the world.

So, in short, mindfulness meditation consists of contemplating the four satipatthanas or foundations of mindfulness: body, feelings, mind, and dhammas (often rendered as "mental objects"); and this contemplation should be accompanied by diligence, clearly knowing, and a specific attitude (free from desires and discontent in regard to the world). The sutta also explicates what the four satipatthanas include: Contemplating the body, the first satipatthana, includes being aware of breathing, body posture, body activities, anatomical parts, elements (both of the latter following the understanding at the Buddha's time), and a corpse in decay; the feelings (*vedana*), the second satipatthana, might more aptly be termed *feeling tones* because what is meant here is just the distinction between positive, neutral, and negative feelings tones; emotions proper and other cognitions are part of the third foundation of mindfulness, the mind; and the fourth foundation of mindfulness deals with the dhammas. In this context, rather than mental objects, a more suitable translation might be "mental factors and categories" (Anālayo, 2003, p. 183). The dhammas explicitly addressed in the fourth satipatthana are central building blocks of the Buddhist "theory": the five hindrances, the five aggregates, the six sense spheres, the seven awakening factors, and the four noble truths (for details, see Anālayo, 2003, 2013; Gethin, 2007; Goldstein, 2013).

In mainstream mindfulness interventions, at least in the manualized format, all participants practice the same technique(s) (although in practice there is still some level of flexibility) and undergo prespecified measurements, mostly only before and after the intervention. This is not so in the traditional context. There, in the ideal case, teachers react very flexibly to the student's needs and abilities, for instance, by administering apt treatments and techniques. This application of skillful means (*upaya*) is especially prominent in the Theravada tradition (e.g., Buddhaghosa, 2010) and can be seen in several examples in the sermons of the Buddha, in both the Theravada and the Mahayana tradition (see Gombrich, 2006, p. 17; Laumakis, 2008, p. 60). But it seems that even without the guidance of teachers, many meditators make choices of their own, depending on their experiences with different approaches. In surveys including sizeable samples of experienced meditators, many of them had experiences in different meditative traditions (Matko et al., 2021a; Sedlmeier & Theumer, 2020).

In the traditional context of mindfulness meditation, there is only one goal: nibbana, variously termed awakening, liberation, and enlightenment (among other terms). However, most contemporary meditators do not pursue this goal but instead are motivated to meditate for a multiplicity of reasons other than reaching nibbana (Sedlmeier & Theumer, 2020). Thus, many contemporary meditators might be very interested in interventions that are custom-tailored to their aims and they might be interested in specific measurements that refer to these aims, even if they practice a traditional approach to meditation. In meditation studies, there are, of course, severe restrictions concerning time and commitment, but treating participants as partners and collaborators (instead of as mere subjects) who have a say in what they do might yield sizeable benefits in terms of motivation and positive effects.

Heterogeneity is often high in meditation studies, because meditators differ in many respects. This heterogeneity may obscure effects in group studies because of the large variation of initial values in the dependent measures (and the ensuing large error component in the results). In contrast, this between-participants error component is absent if one concentrates on single meditators instead of groups of meditators, making it easier to detect similar (or dissimilar) effects across participants. However, conventional single-case designs have their limitations when it comes to drawing causal conclusions. Single-case *experimental* designs that use some sort of randomization and allow for examining cause–effect relationships (e.g., Barlow et al., 2009) are an alternative. Especially the multiple-baseline design has already been employed several times in meditation research, as, for instance, in a pioneering study on the effects of meditation (on the soles of the feet) for youths with Asperger’s syndrome, conducted by Singh et al. (2011). This method was also successfully used in a single-case study with individuals with Alzheimer's disease, using a similar design (Singh et al., 2019). Several single-case studies on the effects of mindfulness interventions on disruptive behavior in children and adolescents have been meta-analytically summarized by Klingbeil et al. (2017). Although single-case designs—mostly in nonexperimental settings—have so far been applied primarily in clinical populations, nothing speaks against using them for examining effects on healthy people. An example of such a study in the context of meditation research is May et al. (2014). The authors compared concentration and loving-kindness meditation with respect to two mindfulness subscales, in a university student sample.

Meanwhile, many sophisticated ways of analyzing the results of single-case studies have been established (for an overview see Fingerhut et al., 2021a; Shadish, 2014), complementing traditional visual analysis. Apart from several alternative (mostly nonparametric) kinds of significance tests, suitable effect size measures have been developed for single-case designs (e.g., Pustejovsky, 2019), and these measures can be summarized using meta-analysis (Chen & Pustejovsyky, 2022). Alternatively, if the number of cases is sufficiently high, multilevel models are also an option (Moeyaert et al., 2017). In sum, single-case experimental studies are methodologically sound and allow for drawing causal conclusions, and there are good reasons to prefer them over group studies: They deal better with heterogeneity of all sorts, allow conclusions for small samples, and enable researchers to analyze the time course of effects (Barlow et al., 2009; Kazdin, 2021; Tanious & Onghena, 2021).

In the present study we explored the effects of a traditional mindfulness program by including participants as collaborators who also analyzed their own results, in a multiple-baseline design. We were interested in whether such a training has any positive effects at all and, if so, whether and how individual effects vary across measures and participants.

## Method

### Participants

The study was run in the context of a small seminar on single-case experimental designs for psychology master’s students at the Chemnitz University of Technology, Germany. All sessions took place online, because of COVID-19 restrictions. On the suggestion of the first author (the instructor), the 11 students, three male and eight female, who took part in the seminar (all coauthors) decided to explore the effects of a traditional mindfulness program with themselves as participants. None of the participants had systematic meditation experience.

### Mindfulness Program

The guided training program in the present study was devised by Bhikkhu Anālayo (2018) and the instructions are freely available on the internet (https://www.windhorsepublications.com/satipatthana-meditation-audio/). We decided on this program because of Bhikkhu Anālayo's excellent reputation as a Buddhist scholar and teacher, especially concerning the topic of the satipatthana sutta, and because of the easy availability of the program. The basic pattern of this mindfulness practice is "being in the present, knowing what is happening, and proceeding accordingly" (Anālayo, 2018, Section 12.7). Following his own scholarly studies, Anālayo (2018) suggested using seven objects of mindfulness meditation, with an emphasis on the body (see Table 1). He argued that the mindful contemplation of aspects of the body is the easiest way to practice mindfulness and that the body can be used as an anchor or a grounding to return to. In Steps 2 to 7 of the program, all preceding steps are briefly repeated and meditators are encouraged to always come back to the grounding of the body.

**Table 1:** Summary of Mindfulness Meditation as Taught by Bhikkhu Anālayo

Satipatthana	Object no.	Object of contemplation
Body	1	*Anatomy*: body scans of skin, flesh, and bones
	2	*Elements:* body scans concentrating on solidity (earth), humidity (water), heat (fire), and movement/breath (wind)
	3	*Death:* connecting the breath with life (every inbreath could be the last)
Feelings	4	*Hedonic tones*: Body scans for pleasant, unpleasant, and neutral feelings (hedonic tones), later extended to nonbodily aspects
Mental states	5	*Qualities of the mind*: “how is the mind?”—with or without mindfulness, lust, aversion, delusion, etc.
Dhammas	6	*Hindrances*: What are the conditions that lead to the arising and overcoming of sensual desire, anger, sloth and torpor, restlessness and worry, and doubt?
	7	*Awakening factors*: Recognizing and cultivating mindfulness, investigation, energy, joy, tranquility, concentration, and equipoise

The descriptions are based on those in Anālayo (2018)

### Procedure

After familiarizing themselves with previous meditation research, with a focus on the varieties of mindfulness meditation (several chapters from Sedlmeier, 2022), participants discussed what kind of single-case experimental design to apply and what measurements to take during the study. The discussion resulted in their unanimous decision to use a multiple-baseline design (over individuals) and to measure concentration, mind wandering, decentering, well-being, affect, and emotion regulation, as well as collecting data about their daily meditation practice. They also agreed to their random assignment to one of three baseline lengths: 1, 2, or 3 weeks. Participants settled on assigning the three baseline lengths to four, three, and four participants, respectively (because the overall number of participants, 11, did not allow for equal distribution of participants to the three conditions), and then randomly allocated themselves into these three conditions, relying on the *sample* function of the R statistical language (R Core Team, 2021).

Because of schedule restrictions, the training phase lasted only 6 weeks instead of the 7 weeks suggested by Anālayo (2018). The first author suggested that participants who felt uncertain about contemplating death (third of the seven objects of contemplation; see Table 1) might skip it. Otherwise, participants agreed to practice both contemplations of dhammas (sixth and seventh objects) in the last week. There were weekly online meetings, held separately for the three cohorts, to discuss open problems and questions. Especially the potential problem of expectation effects that might have an impact on self-ratings on questionnaire items was discussed repeatedly, and participants were reminded that there is also always a possibility that an intervention does *not* work.

Participants were encouraged to perform their practice and make all their measurements at more or less the same time of day. Before the beginning of the baseline phase, they were asked to remember that all measurements during the intervention phase should be taken at least 1 hr after the daily practice to minimize short-lived effects of meditation.

### Measures

It was important that the measures used in the present study be comparable to those used in previous research. Therefore, participants agreed on a variety of measures that have been commonly used in meditation research. However, they did not want to spend too much time on daily measurements. For this reason, they opted to respond to specific items for most of the topics covered (and not to full questionnaires). The resulting list of items was thus a compromise between comprehensiveness (of topics covered) and effort (on the side of participants). The items were screened by participants according to whether they found them relevant and if they made sense to them and then selected according to their factor loadings, if possible. If there was no German version of the respective questionnaire, items were translated from the original English version by the participants. Moreover, to simplify measurements, a common scale was used for all items ranging from 1 (*does not apply at all*) to 7 (*fully applies*), irrespective of the original scale. Only the end points of the scales were labeled to make the assumption of an interval scale more justifiable. The answers to all questionnaire items referred to the last 24 hr. Therefore, if necessary, items were slightly reformulated, that is, put into past tense, which is indicated below by italics. For the analysis of all questionnaire measures, mean ratings per (abridged) instrument were used if that made sense to participants. Participants decided against the measurement of trait mindfulness because the respective questionnaires are quite diverse and only partially correspond with how mindfulness is conceptualized in traditional Buddhist contexts (e.g., Feng et al., 2018; Sedlmeier, 2022).

#### Concentration

Participants decided to use a computer-based test for measuring concentration. To avoid ceiling effects (due to repeated application), the test had to have no fixed maximum. One of the participants found such a suitable test, developed by Jacobs (2014). In this test, the numbers 1 to 20 are randomly distributed in a 5 × 4 matrix and the user's task was to click on the numbers in the correct order. The mean time needed in five correct trials is the main result, which we used in our study. Participants noted the average time (in seconds) in an Excel sheet (in which they also recorded the questionnaire measures). To diminish practice effects, the concentration test was completed every other day. All the following measurements were collected on a daily basis.

#### Mind Wandering

Three items (with the highest factor loadings) were chosen from the Mind Wandering: Spontaneous scale (Carriere et al., 2013): 1. “I *found* my thoughts wandering spontaneously.”, 2. “When I *mind- wandered* my thoughts *tended* to be pulled from topic to topic.”, and 3. “It *felt* like I *didn't* have control over when my mind *wandered*.”

#### Decentering

From the items that loaded on Factor 1 (Decentering) of the Experiences Questionnaire (Fresco et al., 2007), we selected the following five: (1) “I *could* observe unpleasant feelings without being drawn into them”; (2) “I *separated* myself from my thoughts and feelings”; (3) “I *had* the sense that I *was* fully aware of what *was* going on around me and inside me”; (4) “I *could* actually see that I *was* not my thoughts”; and (5) “I *was* consciously aware of a sense of my body as a whole.”

#### Well-Being

All 5 items from the WHO (Five) Well-Being Questionnaire (World Health Organization, 1998, p. 25) were used: (1) “I *felt* cheerful and in good spirits”; (2) “I *felt* calm and relaxed”; (3) “I *felt* active and vigorous”; (4) “I *woke* up feeling fresh and rested”; and (5) “My daily life *was* filled with things that interest me.”

#### Affect

To measure positive and negative affect, participants agreed on using 4 items (adjectives) each from the Positive and Negative Affect Schedule (PANAS; Watson et al., 1988). For positive affect, these were "attentive," "determined," "proud," and "excited," and for negative affect, these were "irritable," "nervous," "guilty," and "ashamed."

#### Emotion Regulation

To measure emotion regulation, only 2 items were selected from the Emotion Regulation Questionnaire (Gross & John, 2003), one for the Reappraisal factor ("When I *wanted* to feel less negative emotion, I *changed* the way I was thinking about the situation", and one for the Suppression factor ("When I *was* feeling negative emotions, I *made* sure not to express them").

#### Daily Meditation Practice

The daily questionnaire ended with 5 items about the meditation practice: (1) “I got easily immersed in the meditation today”; (2) “It was easy to meditate”; (3) During the meditation, I was distracted by something”; (4) “I was relaxed”; and (5) “I was awake.”

### Data Analyses

Data were first analyzed by participants themselves and each wrote a short report about the results. Later, groups of participants made comparative analyses for selected parts of the results. All analyses were performed with the R statistical language. For most analyses, we used the flexible *scan* package (Wilbert & Lüke, 2021; see also Wilbert, 2021). Some participants continued their practice after the 6-week training period. To ensure comparability, only the data for the 6-week training are considered in the analyses.

#### Visual Analysis

Traditionally, the most important kind of analysis in single-case designs involves visually examining the values of dependent variables over phases. Because the number of possible single-case figures for each variable is too large for full presentation, we present only selected visual analyses for illustration.

#### Effect Sizes: Tau-U

Conventional effect sizes such as *d* and *r* are suitable for summarizing single-case data only if it can be assumed that measurements are independent and population values are normally distributed (Solomon et al., 2015), which is almost never the case. Meanwhile, as an alternative, effect sizes that rely on the nonoverlap of data are very common in single-case research. One of the most commonly used is Tau*-U* (Parker et al., 2011). Tau*-U* is, however, not one effect size but a family of effect sizes (see also Brossart et al., 2018; Fingerhut et al., 2021b). We chose the version that allows for incorporating the trend in the treatment phase (if there is a “positive” trend—Trend B in the following—effects increase) and for statistically controlling a trend in the baseline phase (Trend A). When should a baseline trend be controlled? The usual answer is if the trend is statistically significant. However, if baselines are short, the statistical power for detecting such trends is low. Therefore, we used two criteria: We controlled for baseline trend if either the trend in the baseline was statistically significant (α = 0.05) or the Tau for the baseline phase was ≥ 0.50. If no baseline trend could be detected, we used the A vs. B + Trend B version of Tau*-U* in the scan package and otherwise A vs. B + Trend B – Trend A. Note that nonoverlap measures such as Tau-*U* are not directly comparable to conventional effect sizes, although they might be regarded as being roughly comparable to correlations. As yet, there is no unanimous agreement on how the size of a given Tau-*U* should be interpreted. We used the tentative benchmarks proposed by Vannest and Ninci (2015), that is, < 0.20: small, 0.20 to 0.60: moderate, 0.60 to 0.80: large, and > 0.80: very large.

#### Meta-Analysis

Apart from Tau*-U*s, the scan package also calculates standard errors for them. These two kinds of information were used to perform (random-effects) meta-analyses over participants for all dependent measures, using the *metafor* package (Viechtbauer, 2010). The potential impact of the items measuring daily meditation practice was considered by including their means (averaged per person) as moderator variables.

#### Visual Clustering

To obtain an impression of differential effects across participants, we used multivariate plots across the dependent measures (function *stars* in R).

## Results

In the following, the main results are summarized (additional results can be found in the supplementary material for this article). There was no attrition over the whole period and although the training period covered the Christmas and New Year holidays, there was no systematic decline of meditation practice and data collection even in this period.

### Daily Meditation Practice

**Fig. 1:**
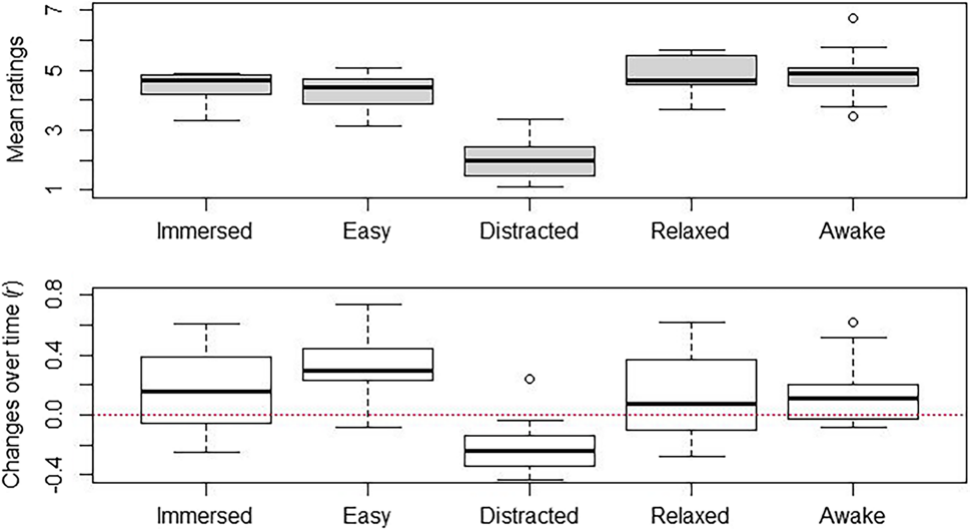
Mean ratings (top) and changes over time (*r*, bottom) for the five measures of the daily meditation practice (1 = *does not apply at all*; 7 = *fully applies*)

Figure 1 shows both the mean ratings for the respective five items (top) over the 6 weeks of the intervention and the change in these ratings over time (bottom), that is, the correlation between measurement point and rating. A positive correlation indicates that scores tended to increase over time and a negative correlation that they decreased. It is evident that on average, participants had a positive impression of their meditation practice (Fig. 1 top) and that they, at least in tendency, became more immersed, found the practice increasingly easy, and were less distracted and more relaxed and awake as their practice progressed over time (bottom). It turned out that two pairs of these five items were strongly correlated: "I got easily immersed in the meditation today" and "It was easy to meditate" (*r* = 0.93); and “I was relaxed” and “I was awake” (*r* = 0.79). Therefore, in further analyses, we used the average of the values of these two pairs of variables to avoid potential multicollinearity and to limit the number of dependent variables somewhat.

### Concentration

**Fig. 2:**
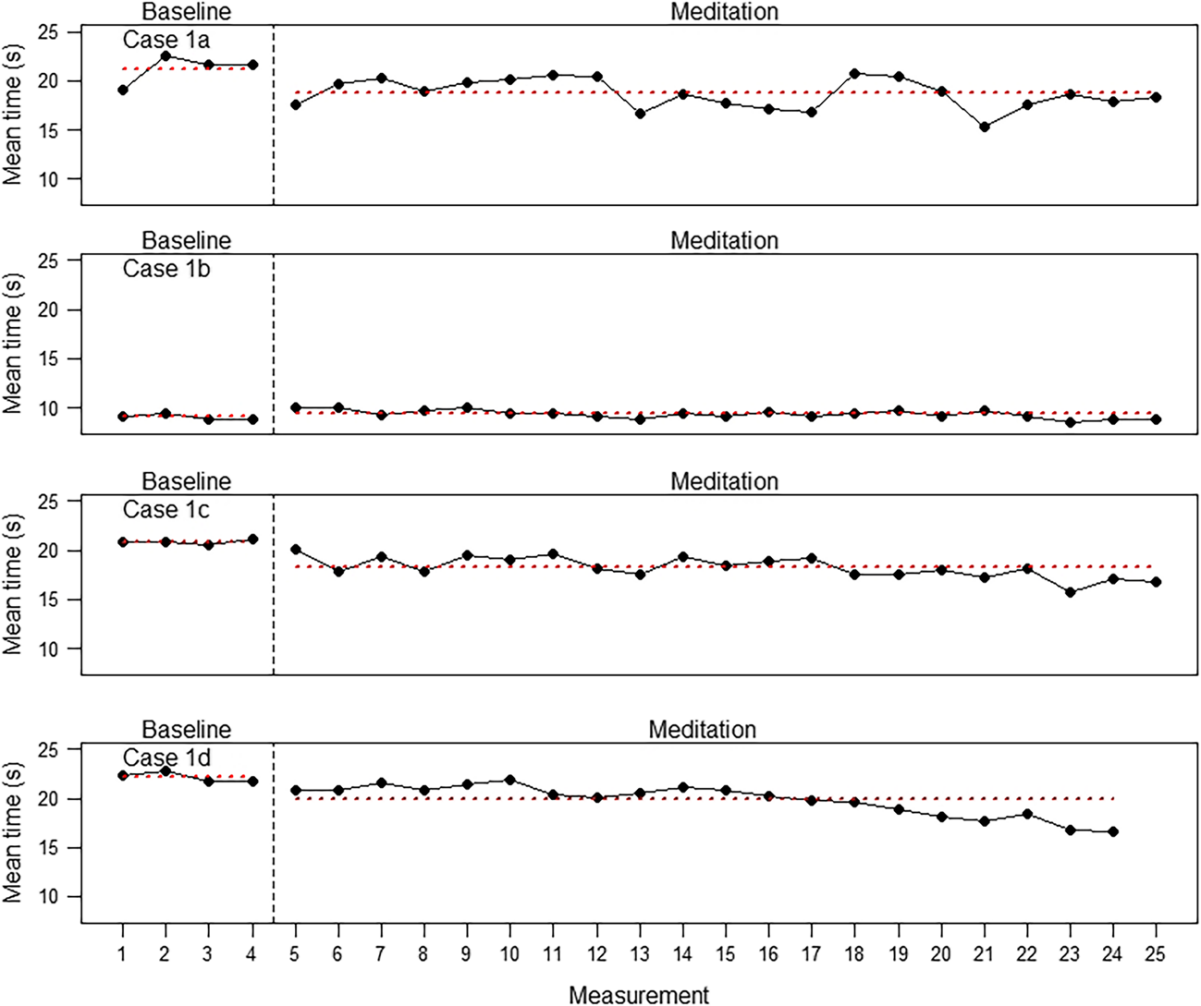
Results for the concentrations task (mean time needed to click on the 20 randomly distributed numbers in the correct order) in five correct trials for the four participants in the 1-week baseline phase

If mindfulness practice improved concentration, the visual analysis should reveal effects, that is, a systematic reduction of the mean time needed to correctly click on the randomly distributed numbers 1 to 20, irrespective of baseline length. For want of space, we first present the results for the four participants with the shortest baseline phase of 1 week (Fig. 2).

**Fig. 3:**
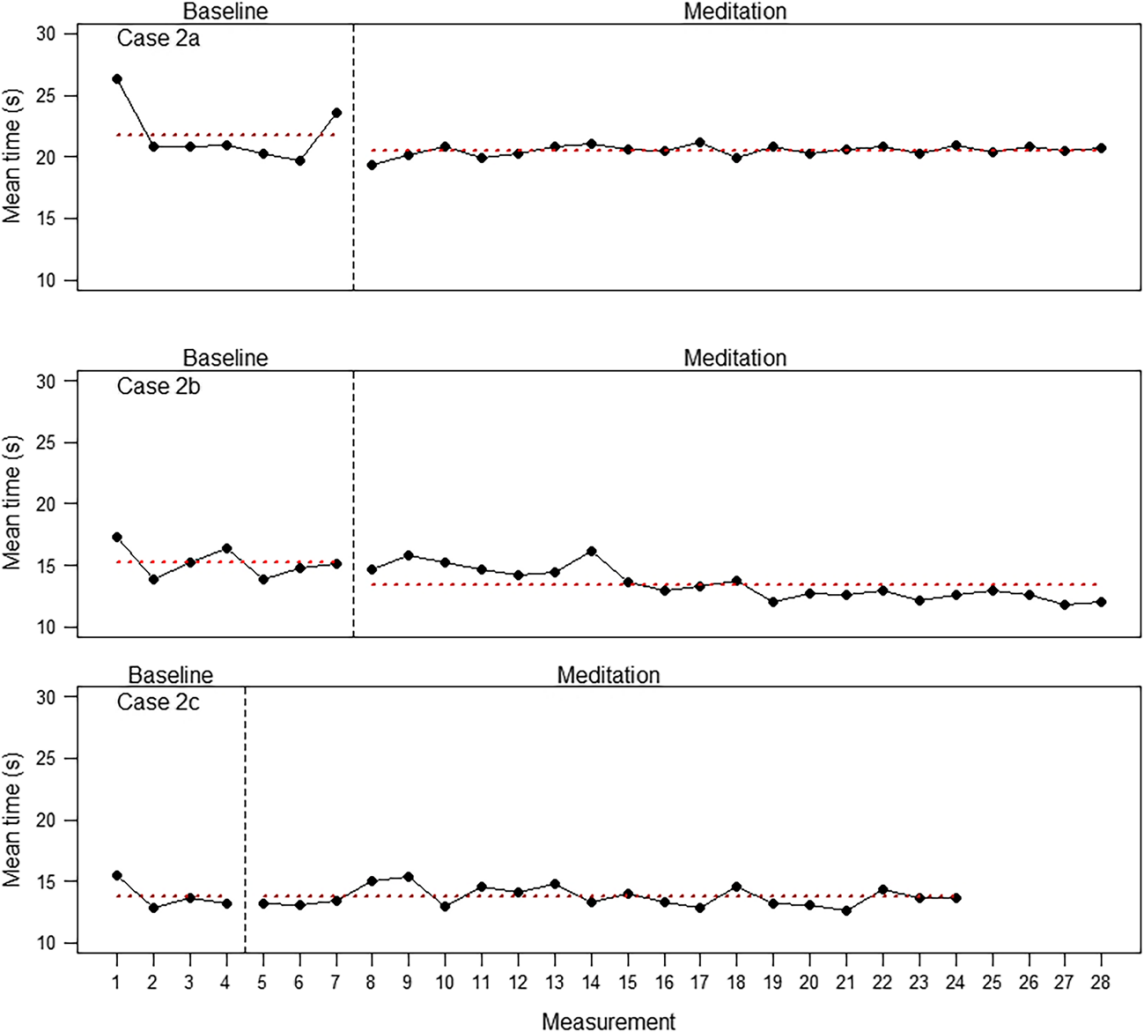
Results for the concentrations task (mean time needed to click on the 20 randomly distributed numbers in the correct order) in five correct trials for the three participants in the 2-week baseline phase. Note that Case 2c did not have internet access during the 1st week and therefore has fewer baseline measurements for this task

Figure 2 indicates a systematic improvement in concentration after the onset of meditation for three participants (Cases 1a, 1c, and 1d) but not for Case 1b. The respective Tau-*U*s were -0.35, -0.65, and -0.77 versus 0.19 (for Cases 1b and 1d, baseline trends were statistically controlled). However, it seems that Case 1b was already very fast from the beginning—with a mean of 9.1 s, a result that might have been hard to improve on. How about participants with a 2-week baseline phase? Figure 3 again indicates slight improvements for two participants, Cases 2a and 2b, although the baseline for Case 2a exhibits a large variance. Case 2c—again the fastest one in the cohort—did not show an improvement. The Tau-*U* for Case 2a does not conform with the visual impression (-0.03) but the other two do (-0.68 and -0.07 for Cases 2b and 2c, respectively).

**Fig. 4:**
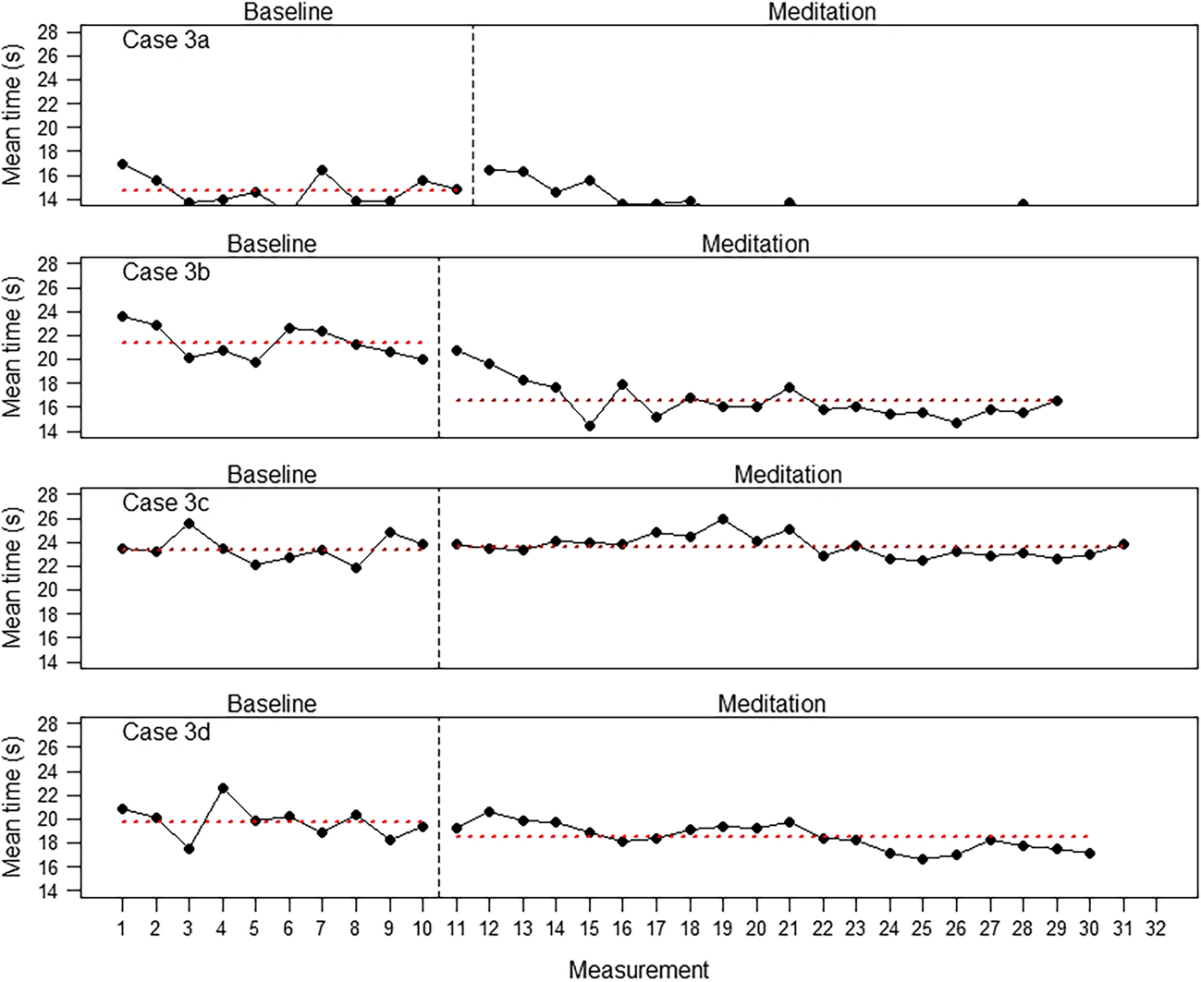
Results for the concentrations task (mean time needed to click on the 20 randomly distributed numbers in the correct order) in five correct trials for the four participants in the 3-week baseline phase. (Note that Case 3a began immediately with the concentration task and therefore has one additional measurement during the baseline phase)

If the improvement in concentration was due solely to practice effects, the size of these effects (difference between the baseline and meditation phases) should be smallest for the longest baseline of 3 weeks. Figure 4 shows that this was not the case. Although, again, one of the participants (Case 3c) did not show improvement, the other three did to varying degrees. The Tau-*U*s were -0.61, - 0.68, -0.06, and -0.54, for Cases 3a, 3b, 3c, and 3d, respectively (for Case 3b, the baseline trend had to be corrected).

**Fig. 5:**
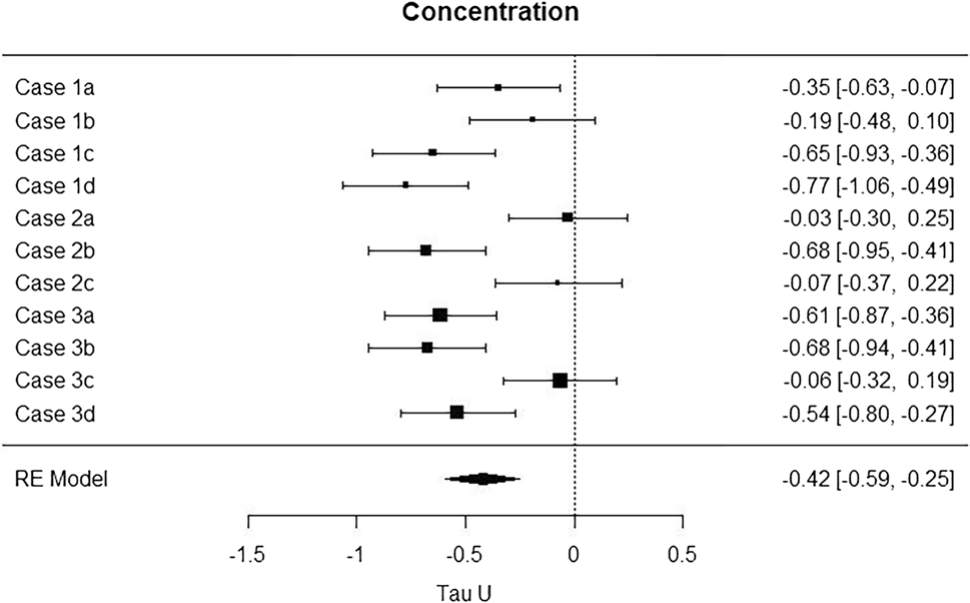
Forest plot of the effect sizes (Tau-*U*) for the concentration task. Shown are means and 95% confidence intervals

A meta-analysis of the resulting Tau*-U*s for the 11 participants yielded a (weighted) mean Tau*- U* of -0.42, 95% confidence interval (CI) [-0.59, -0.25], with values for participants ranging from -0.03 (Case 2a) to -0.77 (Case 1d). Figure 5 shows a forest plot, that is, Tau*-U*s and 95% CIs for each participant (also given as numbers in the rightmost column). It is evident that seven of 11 effects (64%) yielded significant results (confidence intervals do not include zero) and none of the effects were in the unexpected direction. However, a substantial amount of variation was not accounted for (*I*^2^ = 75.80%, *T* = 0.25; *I*^2^ estimates the proportion of heterogeneity attributable to variance in true effects [and not to sampling error], and *T* is an estimate of the standard deviation of the mean population Tau*-U*; see Higgins & Thompson, 2002). When the items that describe participants' perception of their daily meditation practice (mean of "Easy" and "Immersed"; mean of "Awake" and "Relaxed"; and "Distracted") were inserted as moderators, this unexplained variance was considerably reduced (*I*^2^ = 47.93%, *T* = 0.135). The easier the practice was and the more immersed participants were in the practice, the better they could concentrate (*p* = 0.0004). However, the more awake they felt and the more relaxed, the weaker their concentrations was (*p* = 0.006). Whether they felt distracted or not apparently did not have an impact on their concentration.

In sum, meditation on average increased participants' concentration. However, four of the 11 participants did not profit reliably and this might at least in part have been due to how well participants came to terms with different aspects of their daily practice.

### Questionnaire Measures

**Fig. 6:**
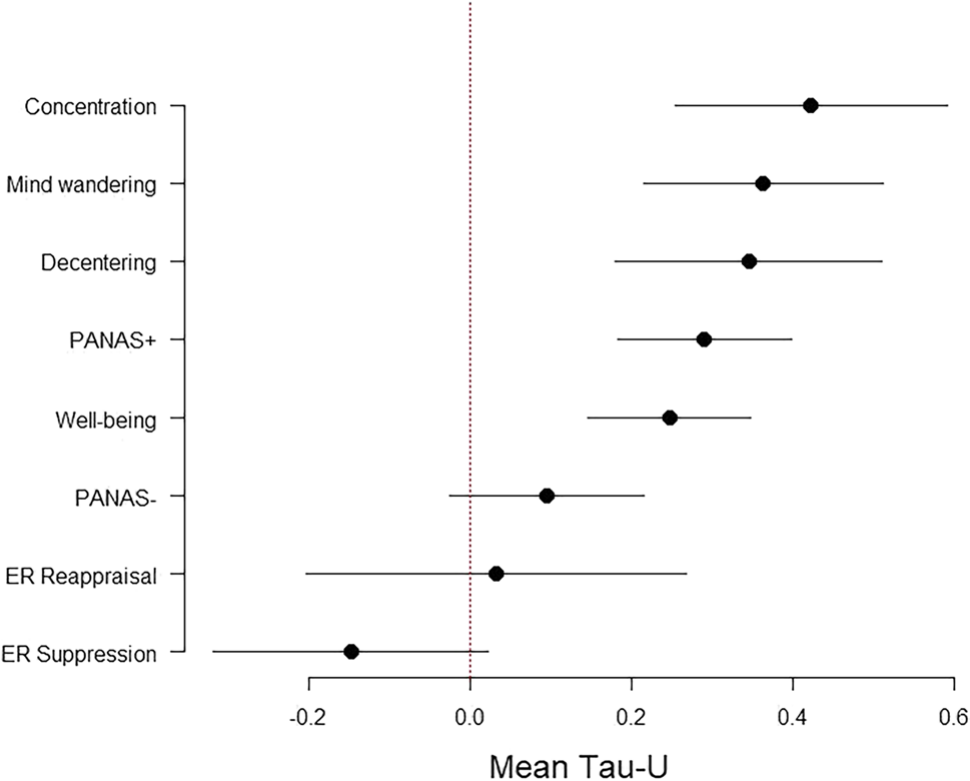
Confidence intervals (95%) for the mean effects sizes (Tau-*U*) of the dependent measures. PANAS+ = mean of four items from the Positive Affect scale of the Positive and Negative Affect Schedule (PANAS; Watson et al., 1988); PANAS- = mean of four items from the Negative Affect scale of the PANAS; ER_Reappraisal and ER_Suppression = one item each from the Reappraisal and the Suppression factor of the Emotion Regulation Questionnaire (Gross & John, 2003)

Results for the questionnaire measures are not presented in full detail for want of space (for forest plots, see the supplementary material). Figure 6 shows a summary of the meta-analytic results (with 95% CIs) for all questionnaire measures and the concentration task, ordered according to (weighted) mean effect size (see also Table 2).

**Table 2:** Summary of the Meta-Analytic Results

Measure	Mean Tau*-U*	95% CI		Percentage of participants with significant results	*I*^2^	*T*	*I*^2^ (moderators included)	*T* (moderators included)
		*LL*	*UL*					
Concentration	0.42	0.25	0.59	64%	75.80	0.25	47.93	0.14
Mind wandering	0.36	0.22	0.51	64%	83.69	0.23	85.03	0.24
Decentering	0.34	0.18	0.51	73%	87.65	0.26	88.63	0.28
PANAS+	0.29	0.18	0.40	82%	69.89	0.15	63.45	0.13
Well-being	0.25	0.15	0.35	73%	66.89	0.14	76.22	0.18
PANAS-	0.09	-0.03	0.22	27%	74.86	0.18	81.07	0.21
ER_Reappraisal	0.03	-0.20	0.27	27%	93.00	0.38	94.17	0.43
ER_Suppression	-0.15	-0.32	0.02	18%	86.80	0.27	81.16	0.22

*Note*. *I*^2^ and *T* are measures of heterogeneity (*I*^2^ is the unexplained variance of Tau*-U*s *not* due to sampling error, and *T* is the standard deviation of the mean Tau*- U*s), shown without and with inclusion of three moderator variables (Easy-Immersed, Awake-Relaxed, and Distracted). *CI* Confidence interval, *LL* lower limit, *UL* upper limit, *PANAS+* mean of four items from the Positive Affect scale of the Positive and Negative Affect Schedule (PANAS; Watson et al., 1988), *PANAS-* mean of four Items from the Negative Affect scale of the PANAS, *ER* Emotion Regulation Questionnaire (Gross & John, 2003)

The concentration task yielded the highest mean effect, followed by mind wandering, decentering, mean positive affect (PANAS+), and well-being. Negative affect and the two items that measured emotion regulation (reappraisal and suppression) did not show any noticeable effect. Including the mean measures for daily meditation practice as moderators in the meta-analyses did not systematically decrease unexplained variation. Apart from the inconsistent effects of these measures on concentration, only positive emotions (PANAS+) increased with increased ease and immersion in the practice (*p* = 0.028). The inconsistent impact of the felt daily meditation practice (also the overall Q-tests did not reach significance, except for concentration and PANAS+) becomes evident in the comparison of *I*^2^ and *T* without and with the three moderator variables included (see the four rightmost columns in Table 2). Indeed, for some dependent measures, heterogeneity even increased if these additional variables were added to the analysis (possibly a slight suppression effect).

If the average subjective daily experience of how easy and motivating the practice was did not have a strong impact on the size of meditation effects, differences in personalities, predilections, and fit between personality characteristics and the demands of the current meditation practice might be probable candidate explanations. We have not collected data that could explain the high heterogeneity found in the meta-analyses but if such a heterogeneity exists it should show in systematic differences in meditation effects across participants.

### Differential Effects Across Participants

**Fig. 7:**
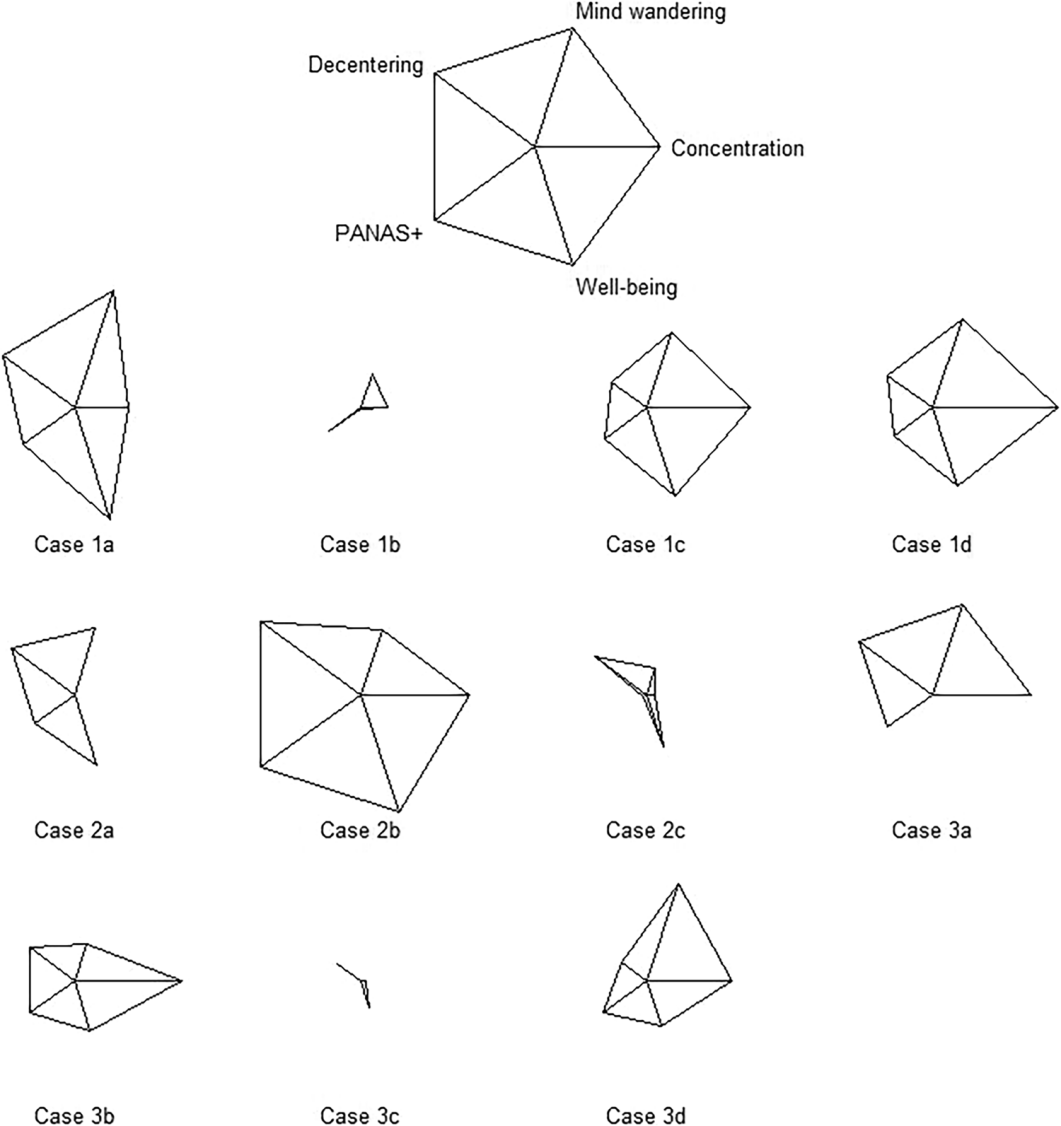
Differential meditation effects (Tau-*U*s) across participants, depicted as star plots. PANAS+ = mean of four items of the Positive Affect scale of the Positive and Negative Affect Schedule (PANAS; Watson et al., 1988)

The two measures for emotion regulation consisted of only one item each and therefore might not be considered very reliable. Therefore, we excluded them when examining differential effects across participants. Also negative affect (PANAS-) might not be a good measure for comparing participants because it already showed a very low mean value in the 1st week of the baseline phase (*M* = 2.21)—indicating that participants overall had little negative affect and so little space to improve—whereas all other questionnaire measures had mean values around the mean of the common scale (*M* = 4). Using the Tau*-U*s for the remaining measures, concentration, mind wandering, decentering, positive affect (PANAS+), and well-being, we constructed star plots (Fig. 7). Note that star plots use standardized values and therefore the lengths of the different rays of the "star" do not allow for absolute but only relative comparisons. Nonetheless, Fig. 7 reveals that participants profited quite differentially from the training and might be tentatively grouped into three clusters. Whereas Cases 1a, 1c, 1d, 2b, 3b, and 3d had gains in all five measures, three participants (Cases 1b, 2c, and 3c) exhibited, in comparison to the others, rather small effects, and two participants (Cases 2a and 3a) exhibited the smallest effect in one measure each (concentration and well-being, respectively). This comparison indicates that traditional mindfulness meditation did not have uniform effects and might not suit everybody.

## Discussion

The present study aimed to explore whether a traditional mindfulness meditation program yields substantial positive effects and, if so, whether the effects are uniform across measures and participants. To examine these questions, a single-case experimental design was used and participants were regarded as collaborators.

Although not all participants improved on all the measures, and not all participants showed improvements to the same extent, the overall picture allows for concluding that a traditional mindfulness training yields beneficial effects similar to those of secular mindfulness programs. Interestingly, the measure that was least prone to bias due to expectation effects (not much cognition involved)—the concentration task—yielded the highest mean effects, followed by two more cognitive measures, mind wandering and decentering. As yet, there do not seem to exist generally accepted criteria for interpreting the size of single-case effect measures, such as Tau*-U*. However, if one uses the benchmarks for Tau*-U* proposed by Vannest and Ninci (2015) our results may be considered roughly comparable to those found in group studies with healthy participants (Sedlmeier et al., 2012, 2018). These meta-analyses, apart from revealing mainly medium-sized effects, report strong variations in effect sizes over different kinds of measurements, a result we also found in the present study. In addition, we could clearly demonstrate that the effects varied considerably across participants. This variation is seldom highlighted in group studies and is not reported at all in meta- analyses of such studies, except indirectly, of course, for the fact that heterogeneity of measurements across participants decreases standard effect sizes (e.g., with a given mean difference between groups, the larger the heterogeneity of effects of group members, the larger the standard deviation of these effects and the smaller the corresponding effect size). The large heterogeneity across participants we found cannot be explained by sampling error alone as indicated by the high scores for *I*^2^ and *T* in Table 2. Therefore, summary results reported in meta-analyses that rely only on group effect measures might severely underestimate the amount of unexplained variance (and the mean effects reported there might be hard to interpret).

What might account for these heterogeneous effects? One candidate explanation might be how the daily meditation practice was perceived by participants. There is some indication that whether participants became immersed in the practice, found it easy to perform, or were distracted, relaxed, or awake might have had some impact (on concentration and PANAS+, in our case). Interestingly, this impact might also depend on what kind of dependent measure one considers. Overall, however, the perception of the daily meditation practice did not make much of a systematic difference, in our analyses. Future studies need to address more potentially intervening factors such as meditators' motivations (Sedlmeier & Theumer, 2020) and personality characteristics (de Vibe et al., 2015; Noone & Hogan, 2018; Winning & Boag, 2015). There are indeed indications that individuals prefer meditation techniques that are compatible with their personalities and also tend to have the highest gains when practicing those techniques (Tang & Braver, 2020).

In our study, participants were regarded (and regarded themselves) as collaborators. After deciding on the effects of meditation as their research topic, they had some choice in selecting the type of meditation (there was also the option of choosing some secular meditation technique, such as a variant of body scan, loving-kindness meditation, open-awareness, or a concentrative technique), they were given some say in deciding on a suitable study design, and they had complete control over the selection of dependent measures. Obviously, we cannot say much about the differences between collaborators and conventional participants concerning the size of meditation effects for want of an apt comparison. But did collaboration raise motivation? We have not used any formal measures, but zero attrition even over the Christmas and New Year holidays, as well as no systematic decline in meditation practice and data collection even in this period, suggests a very high motivation to complete the training. Moreover, group discussions indicated high commitment throughout the study. Of course, this study cannot serve as a general blueprint because many meditators may lack the methodological skills needed to perform extensive data analyses on their own results, which the participants in the current study did possess. However, it should not be a great problem for many meditators to collect daily measurements and make simple graphical analyses (with a little help).

In this study, participants fully collaborated in all phases and we argue that this, apart from being consistent with tradition, might have had beneficial effects. However, our study does not provide empirical support for this claim, except for the observation that the motivation of participants was very high throughout the study. Benefits of participants as collaborators versus as "normal” participants could, however, be examined easily if, for instance, a study designed by collaborator participants was also run with "normal” participants. The benefits of regarding study participants as collaborators can be expected to be stronger when working with experienced meditators (vs. with novices as in the present study). This kind of collaborative research has already been practiced in qualitative studies (e.g., Ataria et al., 2015; Boyle, 2015; Eberth et al., 2019; Full et al., 2013). Especially promising might be a collaboration of meditators who also do meditation research themselves.

## Limitations and Future Research

In our study, we modified existing questionnaires in two respects. First, in most cases, we used only selected items, and second, we employed only one common scale with values ranging from 1 (*does not apply at all*) to 7 (*fully applies*). Obviously, in this case, norms for existing questionnaires do not apply. When using selected items, we took pains to choose the most central ones, as indicated by their factor loadings. This may have had an impact on the modified questionnaires’ validity and reliability. In any case, because of the high number of repeated measurements, conventional questionnaires cannot be utilized without restrictions in single-case experimental designs, with the exception of the initial measurements that might be taken for interindividual comparisons and for use as moderator variables. For intraindividual comparisons—for example, baseline versus treatment—the absolute size of the numbers is not important. However, in addition to practice or mere-exposure effects, there is often the problem of floor or ceiling effects. An example could be the small changes we found concerning negative affect. Because of the already very low baseline values (small negative affect) there was little room for improvement (even lower values). One possibility for dealing with limited scale values that needs to be explored in future studies of this kind might be to have participants construct their own scales and begin with a value of 0. If participants feel that things deteriorate (using the 0 as an anchor), they can choose negative values, and in the opposite case positive ones. If the range of the scale is open-ended, relative judgments can be expected to be valid irrespective of the initial values chosen. Because all comparisons are within, it would not be a problem if the ranges of the resulting scales differed across participants. It is, however, still an open question how well participants come to terms with such scales. And for comparisons across several single-case experimental studies, it would definitely help to reach a consensus about the kinds of measures to be used, including important moderator variables.

Obviously, relying solely on our data, we do not have an answer to the question of what kind of person with what goal and motivation might benefit strongly or not so much from a traditional mindfulness practice. Note that mindfulness practice (*samma sati*) is only one part of the Buddhist Noble Eightfold Path (for a quick introduction, see Rahula, 1959), and there are voices arguing that mindfulness-based interventions should reintegrate the original ethical and spiritual context (Shonin et al., 2015; van Gordon & Shonin, 2020). Indeed, recent research suggests that adding ethical components to the meditation practice might yield additional benefits, possibly moderated by personality factors (Bayot et al., 2020; Chen & Jordan, 2020; Matko et al., 2021b). Future research should include taking a closer look at the impact of ethical and spiritual context by comparing the effects in studies that differ only in that respect. For that purpose it would be worthwhile to also include (new) measures derived from traditional approaches (e.g., Sedlmeier & Srinivas, 2016, 2021). But even if would-be meditators do not find traditional (or contemporary) mindfulness practices helpful, they have a choice of a multitude of other approaches to meditation that might fit their needs better (Dahl et al., 2015; Matko & Sedlmeier, 2019).

In our view, single-case experimental studies might be a good means to deal with the potentially large number of variables that might have an impact on whether would-be meditators benefit from meditation. A large number of single-case experimental studies that also include indicator variables such as personality measures and other explanatory variables could be a very potent basis for custom-tailoring meditation techniques for specific individuals and specific purposes. Instead of making recommendations for a specific person from mean results, results from single-case studies for which the values of indicators variables match those of that person could be used to recommend a specific approach. Apart from this more pragmatic benefit, results of single-case studies can shed more light on the time course of meditation effects and also on systematic differential effects depending on the kind of meditation technique, the personalities and goals of meditators, and other potentially interesting variables. Thus, it also seems worthwhile to intensify the use of single- case experimental design to work on a comprehensive theory of meditation in a bottom-up way.

## Supplementary Information

ESM 1 (PDF 485 kb)

## Data Availability

The data for the study are available from https://osf.io/sk7hf/.
