# Supplementary material for: Mindfulness Meditation According to the Satipatthana Sutta: A Single-Case Study With Participants as Collaborators
Source: Mindfulness (N Y). 2023 Jun 10:1–14. Online ahead of print. doi: 10.1007/s12671-023-02160-1 (PMC10256959; doi:10.1007/s12671-023-02160-1)
Supplement: Supplementary file 1 — (PDF 485 kb) [file 12671_2023_2160_MOESM1_ESM.pdf]

Supplementary material: Results of the meta-analyses for the questionnaire items (forest plots with confidence intervals)

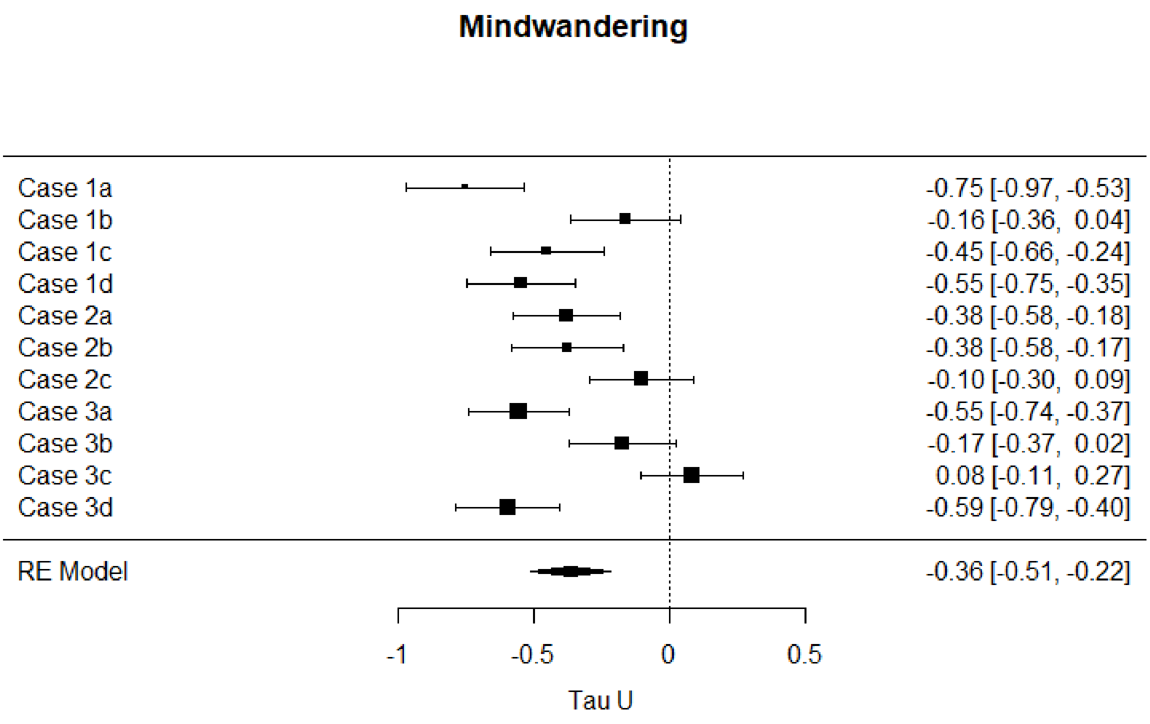

Figure S1. Forest plot of the effect sizes (Tau-U) for mindwandering.

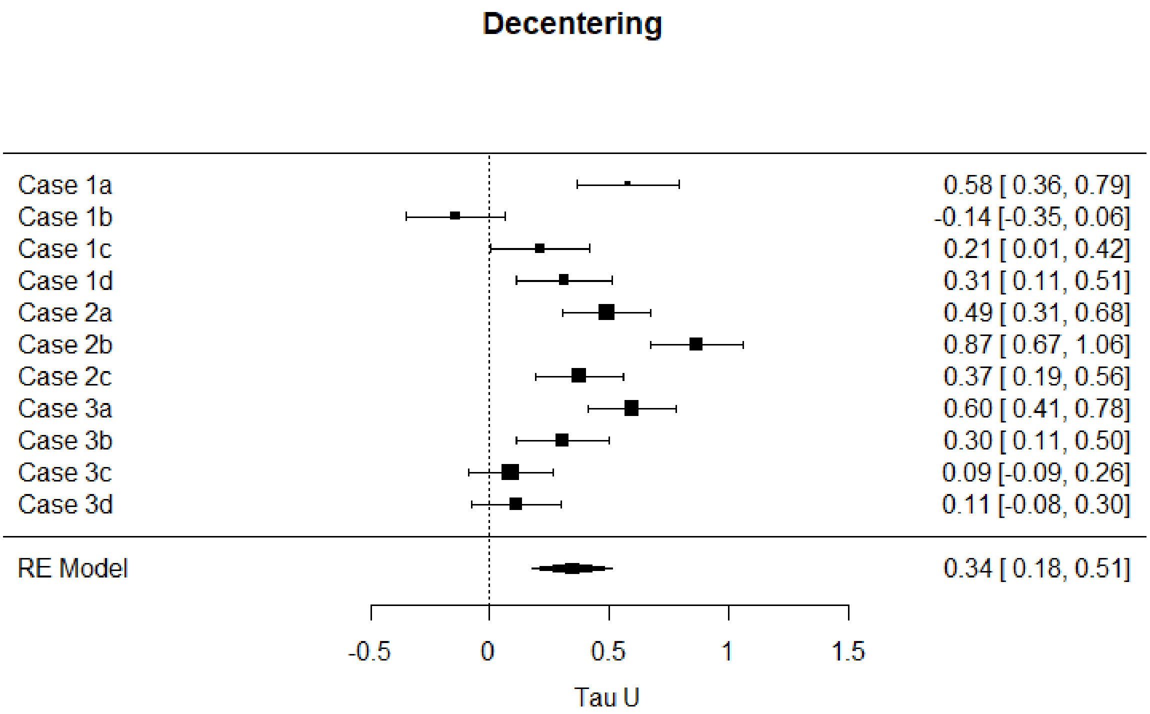

Figure S2. Forest plot of the effect sizes (Tau-U) for decentering.

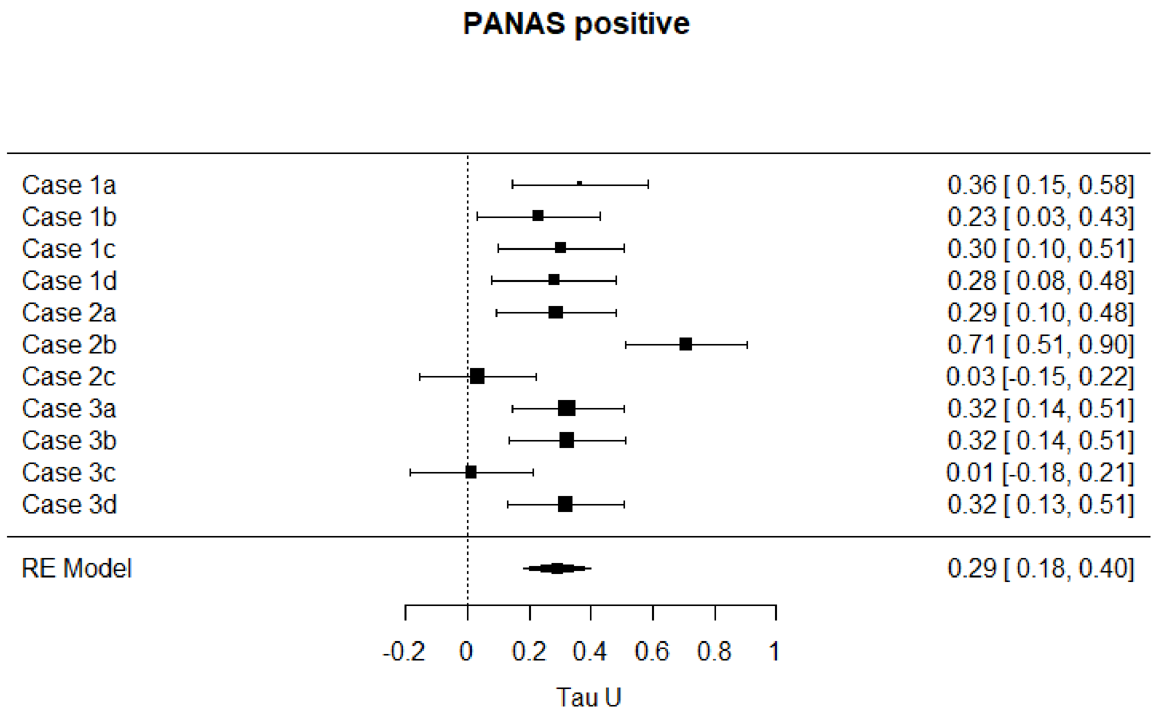

Figure S3. Forest plot of the effect sizes (Tau-U) for PANAS+.

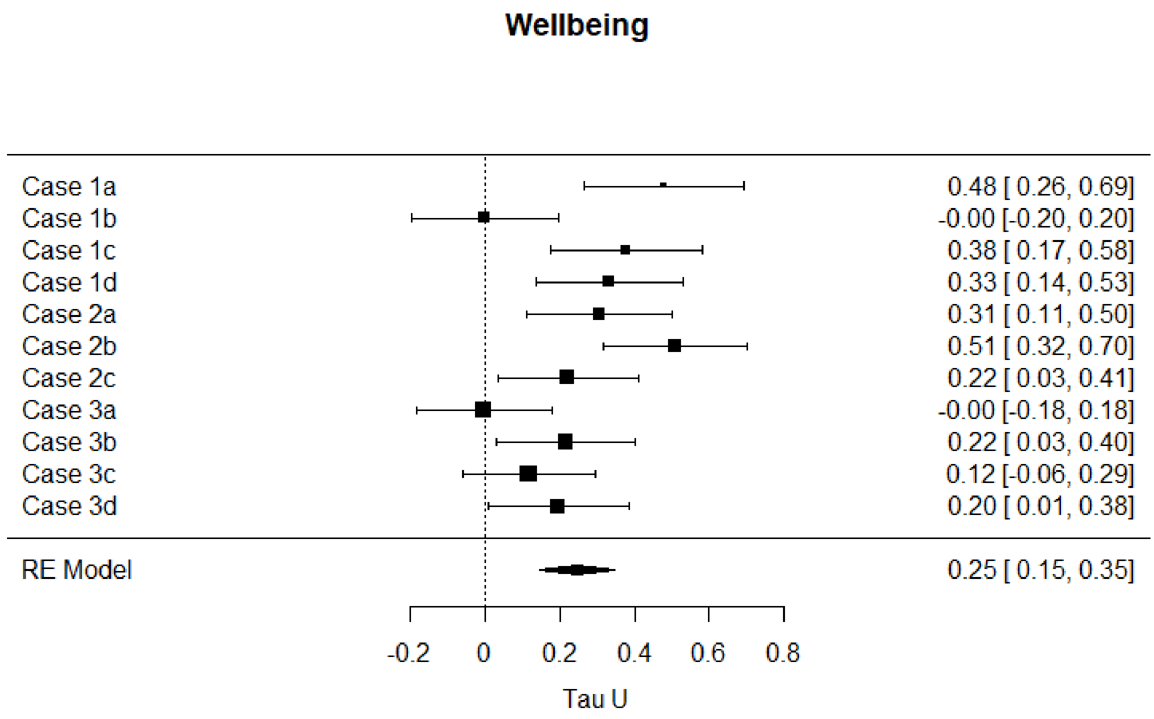

Figure S4. Forest plot of the effect sizes (Tau-U) for well-being.

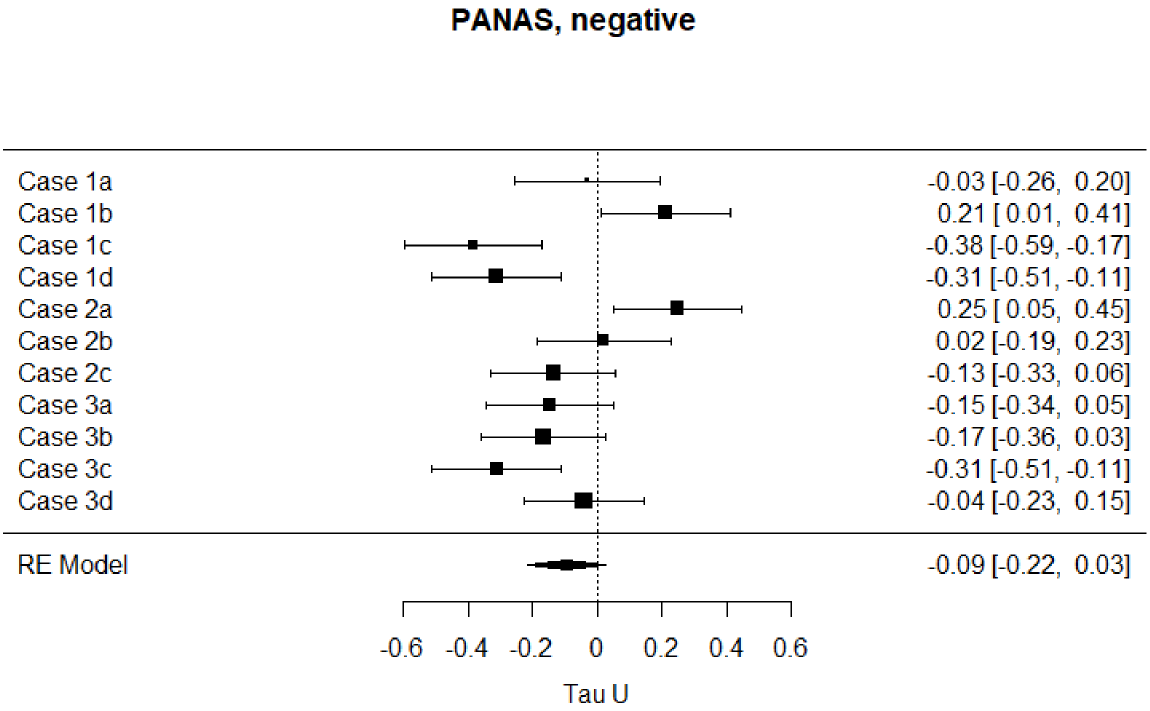

Figure S5. Forest plot of the effect sizes (Tau-U) for PANAS-.

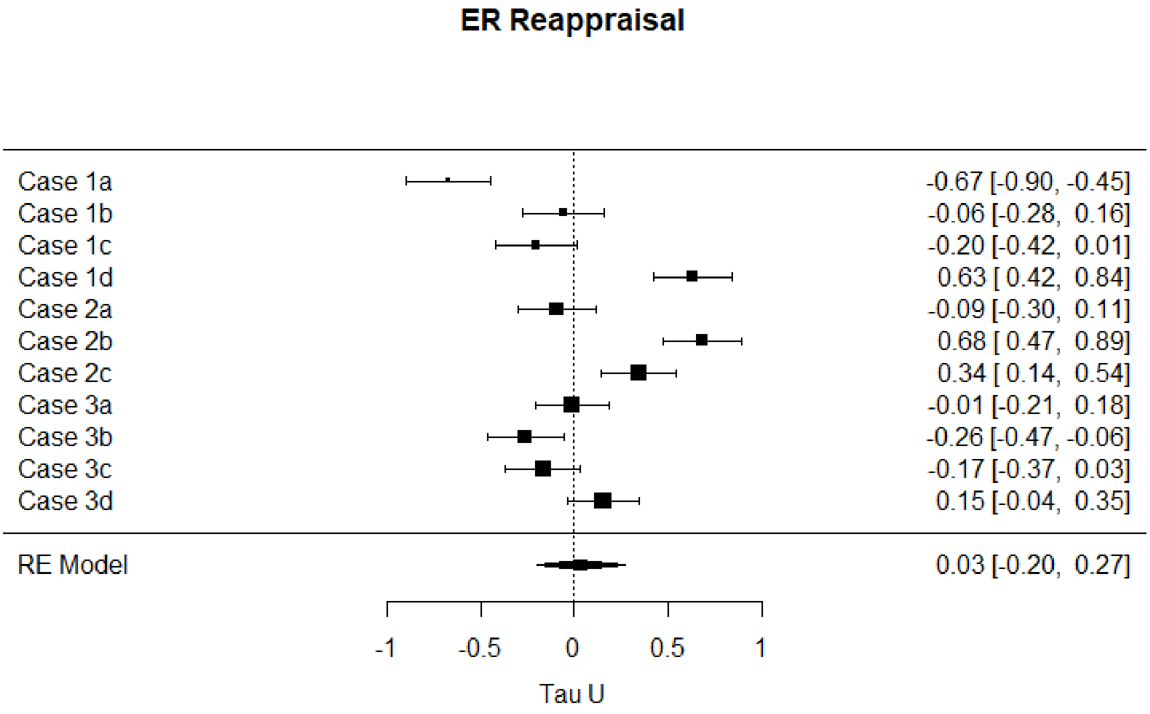

Figure S6. Forest plot of the effect sizes (Tau-U) for ER reappraisal.

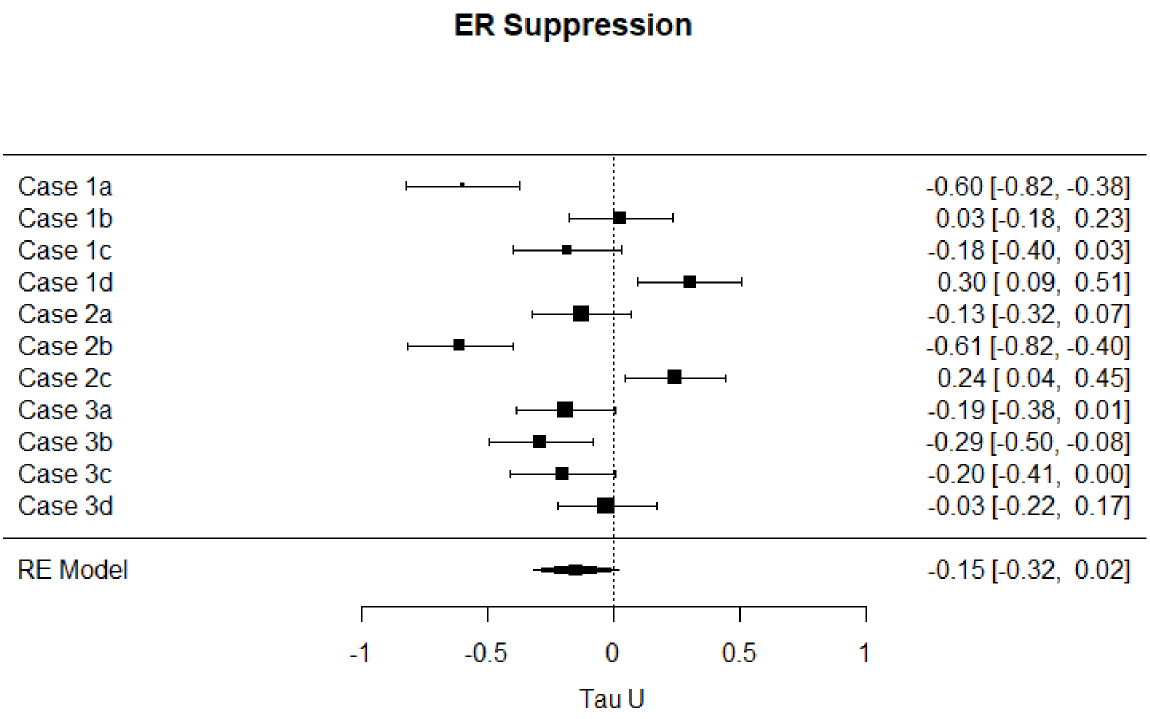

Figure S7. Forest plot of the effect sizes (Tau-U) for ER suppression.
